# Pesticide Residues on Three Cut Flower Species and Potential Exposure of Florists in Belgium

**DOI:** 10.3390/ijerph13100943

**Published:** 2016-09-23

**Authors:** Khaoula Toumi, Christiane Vleminckx, Joris van Loco, Bruno Schiffers

**Affiliations:** 1 Gembloux Agro-Bio Tech/ULg—Laboratoire de Phytopharmacie, Passage des Déportés 2, Gembloux 5030, Belgium; bruno.schiffers@ulg.ac.be; 2 Institut Scientifique de Santé Publique, OD Food, Medecines and Consumer Safety, Rue Juliette Wytsman 14, Brussels 1050, Belgium; Christiane.Vleminckx@wiv-isp.be (C.V.); Joris.vanloco@wiv-isp.Be (J.v.L.)

**Keywords:** cut flowers, roses, pesticide residues, exposure risk evaluation, florists

## Abstract

In order to assess the prevalence of pesticide contamination and the risk of florists’ exposure when handling cut flowers, sampling and analysis of 90 bouquets of the most commonly sold cut flowers in Belgium (50 bouquets of roses; 20 of gerberas, and 20 of chrysanthemums) were carried out. The bouquets were collected from 50 florists located in the seven largest cities of Belgium (Antwerp, Brussels, Charleroi, Ghent, Leuven, Liege, and Namur) and from five supermarkets located in the different regions. To have a better understanding of the route of exposure and professional practices a questionnaire was also addressed to a group of 25 florists who volunteered to take part in the survey. All florists were interviewed individually when collecting the questionnaire. The residual pesticide deposit values on cut flowers were determined in an accredited laboratory using a multi-residue (QuEChERS Quick Easy Cheap Effective Rugged Safe) method and a combination of gas chromatography (GC) and liquid chormatograhphy (LC) analysis. A total of 107 active substances were detected from all samples; i.e., an average of about 10 active substances per bouquet. The most severely contaminated bouquet accumulated a total concentration of residues up to 97 mg/kg. Results show that roses are the most contaminated cut flowers; with an average of 14 substances detected per sample and a total concentration per rose sample of 26 mg/kg. Some active substances present an acute toxicity (acephate, methiocarb, monocrotophos, methomyl, deltamethrin, etc.) and exposure can generate a direct effect on the nervous system of florists. Nevertheless, fungicides (dodemorph, propamocarb, and procymidone) were the most frequently detected in samples and had the highest maximum concentrations out of all the active substances analysed. Dodemorph was the most frequently detected substance with the highest maximum concentration (41.9 mg/kg) measured in the rose samples. It appears from the survey that, despite being exposed to high deposits of residues, florists usually do not protect themselves from contact with residues even if they spend several hours handling cut flowers and preparing bouquets (from 2 to 6 h/day, depending on the time of year and/or selling periods) daily. Bad habits (eating, drinking, or smoking at work) and absence of personal protective equipment of most florists also increase the risk of contact with pesticide residues.

## 1. Introduction

Flowers are used for beautification purpose or given as an expression of love, friendship, gratitude, or appreciation [1]. They are sold throughout the year, but with peak periods (Valentine’s Day, Halloween, Mother’s Day, New Year, etc.). Today, the cut flowers world market represents about 30 billion Euros per year. Europe and North America are still the main markets [2]. The European demand of cut flowers (cut flowers and pots) is estimated to 13 billion Euros, representing 50% of the world’s demand [3]. As a result, millions of flowers produced in Africa, India, Israel, or Latin America travel by road and air to consumer markets located essentially in the rich or emerging countries of the Northern hemisphere. Three hundred fifty million cut flowers are imported each year in the United States and similar quantities are imported in Canada and Europe [4].

Flower production is a dynamic sector in European horticulture with a high growth potential and a major economic weight in international trade [5]. Traditionally in Europe, floriculture is most strongly developed in The Netherlands and Belgium, but cut flowers are also among the intensive crops grown in greenhouses in Great Britain [6]. As in any intensive culture, flowers require the use of a wide range of pesticides to control diseases and pests, which can damage production and marketability. Plants and flowers entering into the European market must meet stringent regulations on plant health designed to prevent introduction of some pests or diseases. Therefore, imported cut flowers receive heavy pesticide applications prior to shipment. In 1977, a sampling of all flowers imported to Miami on three consecutive days showed that 18 bouquets of 105 (17.7%) contained pesticide residue levels superior to 5 ppm, and three samples had levels superior to 400 ppm [4]. The lack of maximum residue limits (MRL) for flowers explains that, unlike other crops which are harvested for consumption, there is no restriction on the use of pesticides and cut flowers are often treated regularly up to harvesting or even after harvest. This also explains the modest development of an “organic” sector or integrated pest management (IPM) in floriculture.

Many pesticides applied on flowers are persistent, dislodgeable by contact with the hands, and are fat-soluble. As they can easily be absorbed through skin contact, florists who handle the flowers daily and for several hours can potentially be exposed to residual deposits of pesticides and possibly endanger their health. Health problems have been reported all over the world for workers and professionals exposed to pesticides daily, including contact allergies, dermatitis and skin effects [7,8], neurologic pathologies [9,10,11,12,13] or even increases in certain types of cancers [10,14,15], hematotoxic effects [16,17], endocrine disruptor effects [18], or cytogenetic damage [19]. Hormonal and reproductive problems of workers (abortions, prematurity, stillbirth, and congenital malformations, low fecundity) have also been reported [20,21]. Various detrimental health disorders were mentioned for female florists and their children in Colombia [22,23] and other developing countries. Therefore, in Europe, EFSA (European Food Safety Authority) reviews, in close cooperation with EU Member States, the risk of exposure of each active substance for operators, workers, bystanders, and residents before plant protection products are allowed to be used in crops or greenhouses [24]. Nevertheless, despite an important potential exposure and a subsequent high level of risk for this group of workers, only a small amount of information was available in Belgium or in Europe about the contamination of flowers and the exposure of florists in link with their professional practices. This information is crucial when people want to assess the risk. As a first step in developing an exposure assessment framework of florists (hazard identification and characterization) we have investigated the extent and severity of pesticide contamination (nature, frequency, and concentrations of pesticide deposits) of the most commonly sold cut flowers in Belgium and the main activities of florists to prepare the bouquets. This survey will be completed later by results of field and laboratory trials to measure the dislodgeable foliar residues (DFR), the transfer from plant to hands and, finally, to estimate the dermal exposure of florists to pesticides applied on cut flowers.

## 2. Methods

### 2.1. Sampling of Cut Flowers

In order to assess the prevalence of pesticide contamination and to evaluate the average levels of contamination of the cut flowers most commonly sold in Belgium (roses, the number one flower sold annually, gerberas, and chrysanthemums) a sampling of 90 bouquets (50 of roses, 20 of gerberas, and 20 of chrysanthemums) was carried out at 50 florist’s premises. The sampling size was estimated according to a similar study carried out by Morse et al. [4] who estimated the minimum sample size required to detect 10% of contamination when a 0% level is expected to be 77 samples, and sampled 105 bouquets from 43 different growers to assess flower contamination in the United States.

Fifty samples of roses (at least five stems per bouquet) were collected within three consecutive days in February (the Valentine Day period). The bouquets were sampled from 45 florists located in the seven largest cities of Belgium (Antwerp, Brussels, Charleroi, Ghent, Leuven, Liege, and Namur) and from five supermarkets located in the different regions. Bouquets of gerberas and chrysanthemums were collected in 25 florist’s shops located in Brussels and Wallonia within three consecutive days in April. After collection, the sampled bouquets were kept in a cool room in vases filled with water and two centimetres of stems were cut obliquely using a sterilised sharp knife to maintain water absorption during storage before analysis. Although cut flowers normally last a fortnight in these conditions, the samples were kept for no more than three days before being taken to the analytical laboratory (transport by road from Gembloux to Ghent).

### 2.2. Analysis of the Residual Pesticide Deposits on the Bouquets

The residual pesticide deposits on the bouquets were analysed by PRIMORIS (formerly FYTOLAB, Technologiepark 2/3, 9052 Zwijnaarde, Belgium) laboratory holding a BELAC (Belgian Accreditation Council) accreditation to ISO/CEI 17025 for pesticide residues on vegetables and herbal products in general. PRIMORIS is an independent, accredited, and officially recognized service laboratory. Samples were analysed with a multi-residue (QuEChERS) method validated by the laboratory for analysis of residues in foodstuffs, which will detect approximately 500 different active substances in a single analysis thanks to a combination of gas chromatography (GC) and liquid chromatography (LC). The QuEChERS method is based on work accomplished and published by Anastassiades et al. [25]. After the sample (five flower stems) had been totally crushed, one homogenous 10 g sub-sample is homogenized by vortex mixing in a blender with acetonitrile to extract the residues. After agitation the extract is put through a clean-up column prior to analysis by gas or liquid chromatography with mass spectrometry (GC or LC-MS/MS) according to the active substances to be determined (GC-MS/MS for small, thermally-stable, volatile, non-polar molecules, or LC-MS/MS for larger, thermolabile, non-volatile, and polar molecules). For almost all active substances, the quantification limit (LOQ) was ≤0.01 mg/kg.

### 2.3. Statistical Analyses

All results of pesticide residues (number of active substances (a.s.) found and the total load of pesticides per sample) were analysed with a Student’s *t*-test using Minitab 16 Statistical Software (Minitab Inc., State college, PA, USA).

### 2.4. Exposure Scenario of Florists

EFSA has adopted the following definition for “workers”: they are persons who, as part of their employment, enter an area that has previously been treated with a plant protection product (PPP) or who handle a crop that has been treated with a PPP. Since worker exposures can vary substantially for a given scenario (e.g., nature of activities and duration of work), it is necessary to have a clear idea of the professional practices in order to be confident that individual exposures will not be importantly underestimated. As the sources of exposure are in contact with foliage, exposure of florists must be estimated for activities that involve significant contact with treated plants. To have a better understanding of the route of exposure and professional practices a questionnaire was also addressed to a group of 25 florists who volunteered to take part in the survey. All florists were interviewed individually when collecting the questionnaire. The size of the group was considered large enough to be representative as they all sell the same flowers in Belgium and have the same activities to prepare the bouquets. In a similar study in the United States [4] 20 flower inspectors participated and only 12 were interviewed. The florists were randomly chosen from professionals located in the Province of Namur (16 florists, i.e., 64%), and the Brussels-Capital Region (nine florists, i.e., 36%). They were asked to answer a detailed questionnaire (five pages) on their personal history, the flowers sold from their premises (flower species and origins), their usual practices, their estimated working hours, their personal protective equipment (PPE) worn, their hygiene rules, and their perception of health problems linked with their occupation. All of the questionnaires were filled in and collected in the week during which the samples were taken for analysis of residual pesticide deposits.

## 3. Results

### 3.1. Origins of the Cut Flowers Collected and Analysed

The 25 florists surveyed purchased flowers from wholesalers. The roses had the widest range of sources: 96% of the florists surveyed purchased roses from Holland, 92% from Belgium, 60% from Kenya, 40% from Israel, 36% from Ecuador or Ethiopia and Morocco (12% together). 80% of the florists purchased chrysanthemums from The Netherlands, 72% from Belgium, and 4% from Israel. Gerberas came, in order of importance, from Belgium, The Netherlands, France, and Israel. As the bouquets were sampled randomly at the shops visited, there was no attempt to reproduce the origins declared in the questionnaires proportionally for the samples analysed. During sampling, the bouquets were labelled and the countries of origin identified by asking the florist. The origin of bouquets collected in the supermarkets was unknown. As expected from the survey, declared countries of origin vary widely for roses (eight countries, Belgium included), while gerberas and chrysanthemums collected were identified as flowers from Belgium and The Netherlands, but the traceability of cut flowers cannot be considered as reliable.

### 3.2. Global Results of Analyses of Residual Deposits

The pesticides residues levels (Table 1) and the number of active substances (Table 2) were determined on the 90 samples of cut flowers.

A statistical analysis performed on the results shows a significant difference between contamination levels according to the species (Table 3).

**Figure 1:**
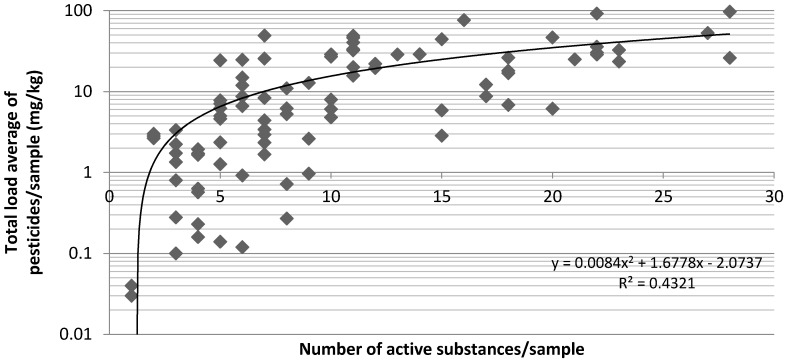
Variation in the total load of pesticides (mg/kg)/sample according to the number of active substances detected/sample.

It appears also that the bouquets on which the highest number of different a.s. have been detected are also those which were the most contaminated by residues. This can be interpreted as an index of bad phytosanitary practices (numerous and repeated treatments with several PPP instead of an alternation between them in an Integrated Pest Management scheme) (Figure 1). Whatever their origins, samples are contaminated by numerous a.s. (22 up to 60 different a.s. according to declared country of origin). A total of 107 a.s. are present (Table 4).

### 3.3. Detailed Results of Analyses: Nature and Prevalence of Pesticide Residues

The following table list the 107 active substances found (concentration > 0.01 mg/kg) in the 90 samples of cut flowers and their maximum concentrations (Table 5 and Table 6). Frequency of a.s. in samples varies between the three species: dodemorph (a fungicide) is the most frequent active substance for roses, fluopyram (a fundicide) for gerberas, and bifenazate, thiamethoxam, and tolclofos-methyl (an acaricide, an insecticide and a fungicide) for chrysanthemums.

Among the 90 flower samples analysed, the highest maximum concentrations out of all the active substances analysed were for dodemorph, propamocarb, and procymidone, with 41.9, 35.4, and 35.3 mg/kg, respectively. Regarding the three species, the highest average concentrations were found:

On roses, for methiocarb, thiophanate-methyl, and furalaxyl (13.60, 9.90 and 8.90 mg/kg, respectively).

On gerberas, for chlorothalonil, flonicamid, and spirotetramat (2.00, 1.71, and 1.37 mg/kg, respectively). Four of the 30 active substances detected in the 20 gerbera samples present maximum concentrations of 2 mg/kg and above. Flonicamid and fluopyram present the highest maximum concentrations, with 3.3 and 3.0 mg/kg, respectively.

On chrysanthemums, for acephate, chlorothalonil, etridiazole, methiocarb, and fluopyram (1.06, 1.21, 1.52, 1.80, and 2.57 mg/kg, respectively). Eleven of the 31 active substances detected in the 20 chrysanthemums samples presented maximum concentrations of 1 mg/kg and more. Tolclofos-methyl, methiocarb, and fluopyram presented the highest maximum concentrations, with 5.6, 6.0, and 6.4 mg/kg, respectively.

### 3.4. Hazard Characterization: Classification of Active Substances According to Their Toxicity

The risk for workers to develop adverse health effects is the combination of health hazards (mode of action; acute and chronic toxicity of a.s.) of pesticides with the likelihood of exposure (concentration levels on flowers; routes of exposure; mitigation measure such as PPE). Both acute and chronic toxicity are of concern for florists. The biological activity is often linked with the toxicity in animals and humans. Insecticides are, in general, the most acutely toxic products, whereas fungicides are considered as less toxic compounds. Many other properties (such as solubility and cutaneous absorption) may interfere with exposure. Of the 107 detected active substances, most belong to groups known for their toxicological properties: organophosphates (12 a.s.); pyrethroids (8 a.s.), and carbamates (7 a.s.) are all pesticides with an action on the nervous system. Since florists are mainly exposed by the dermal route, it was interesting to consider the classification of all a.s. found on cut flowers for acute dermal toxicity according to the CLP(Classification, Labelling and Packaging) Regulation (EC) No. 1272/2008 (Table 7). Most a.s. have a LD_50_ >2000 mg/kg bw and are not classified for that property (86 a.s. of 97 for roses; 27 a.s. of 30 for gerberas; 28 a.s. of 31 for chrysanthemums).

In addition, classification according to CLP regulations for the different health hazards is reported in Table 8. According to this table, the number of sensitizing active substances detected in the roses, chrysanthemums, and gerberas were 16, 11, and 12, respectively.

As the florists handle the flowers every day in the course of their work, the exposure risk is also chronic. The reference value considered for this category of workers is the AOEL (Acceptable Operator Exposure Level) [33] (Table 9). The AOEL is the maximum amount of a.s. to which the worker (in this case) may be exposed without any adverse health effect. It is expressed in mg/kg bw/day.

### 3.5. Lesson Learned from the Florist Observations and Interviews

The great majority (79%) of the questionnaires were filled in by the heads of the businesses. Fifty-six percent of the 25 Belgian florists interviewed were male. Twenty-four percent of the florists were aged between 20 and 30 years, 44% between 30 and 50, and 32% were over 50. Sixty-eight percent of the florists worked alongside other people (employees or family members who are also occasionally exposed). Florist exposure can arise from their activities and can vary according to the working time spent on handling cut flowers. According to the survey, they all have similar activities, such as handling, sorting, pruning, bundling of flowers, and preparation of bouquets. Activities were carefully observed to be repeated later at the laboratory. Sixty percent of the florists worked between 6 and 7 h a day (40% more than 8 h). The time spent preparing bouquets and handling flowers vary greatly over the year, but is always quite high, varying on average from 2 to 6 h a day for 80% of the florists in the low season, and for 40% of the florists in the high season. This handling time could be in excess of 6 h for 8% of the florists in the low season, but during the high season or special occasions, an intense working period, 60% spent more than 6 h a day on this work. Only 12% of the florists worked fewer than 2 h a day in the low season. In addition, the majority of the florists (18 out of the 25 respondents) worked six or even seven days a week. The others worked five days a week. Regarding the potential long term exposure of florists, the survey showed that 44% of the respondents had been working as florists for less than 10 years, but more than 30% had been working as florists for more than 30 years.

With regard to the use of personal protection equipment, 96% of the florists wear no special clothing. Only 20% of the florists surveyed use occasionally latex gloves when preparing bouquets and handling flowers. With regard to hygiene practices, 84% wash only their hands after handling flowers; 20% wash their hands and arms, and 8% their hands, arms, and faces after working. Sixty-five percent wash thoroughly all over after their day’s work. Eighty-eight percent of the florists eat and drink and 12% smoke, during working. None of the florists surveyed use PPP themselves (some used CHRYSAL^®^, an aluminium sulphate, to lengthen the life of the cut flowers).The main routes of exposure during post-application activities are dermal and by inhalation [33]. Inhalation could be later investigated because some pesticides are rather volatile and the plants are stored directly on the premises of the shop where florists are working. This could lead to a significant concentration of active substances in the air. Oral exposure may also occur secondarily to dermal exposure, through hand to mouth transfer. However, for workers, maximum potential exposure by this route is generally assumed to be negligible in comparison with that via the dermal route and by inhalation [33]. Sixty percent of the florists surveyed had not received any information regarding the presence of residual pesticides on cut flowers. Thirty-six percent of them had received information through the media. Only 4% had received information from health workers. With regard to health, four subjects declared that they had eye problems, one declared respiratory problems, and four declared irritations and itching of the skin. Only one florist mentioned headaches and recurrent tiredness. Of the 25 florists surveyed, two suffered from cancers, seen had skin allergy problems, and one suffered from thyroid problems.

## 4. Discussion

From the results of this survey, cut flowers (roses, gerberas, and chrysanthemums) sold in Belgium were found to be heavily contaminated by pesticide residues. The first significant result is the overall contamination of cut flowers. Only a single sample analysed (chrysanthemums from the Netherlands) was free from detectable residues, rather than 16 (15.2 per cent) of 105 lots that did not contain any pesticide residues in the study of Morse et al. published in 1979 [4]. On the contrary, most active substances (a.s.) reached high levels of residues, with concentrations between 10 and 50 mg/kg, about a thousand times above the maximum limit value set for residues in foodstuffs. Sixty percent of flowers had total pesticide residues >5 mg/kg and 4% had concentrations >50 mg/kg.

The second lesson learned from the analyses is the large number of a.s. detected on flowers. No fewer than 107 active substances (almost 10 active substances/sample) were detected in the 90 cut flower samples (roses, gerberas, and chrysanthemums) with a total pesticide load average of 15.72 mg/kg per flower sample. The high pesticide levels on cut flowers are apparently bound to the use of a large number of different pesticides on flowers by growers and can be explained by the pressure of pests and diseases, the lack of alternative pest control methods, the commercial value of flowers which should be perfect at harvest, and the absence of maximum residue limits. The analyses of samples declared of Belgian or Dutch origins reveal the abnormal presence of 15 active substances which are not authorised for use in the EU. These results should, however, be put into perspective as we have no firm guarantee of the origin of the samples taken from the retailer premises rather than from the producers. Nevertheless, the frequency of the presence of active substances not authorised in the EU is significantly higher in the Belgian samples, regardless of the species involved, which could be alarming if flowers were produced in Belgium but, generally, the Belgian official controls do not reflect a misuse of pesticides [35].

Of the three species, roses are the most heavily contaminated by pesticide residues, with an average total load of active substance per sample of 26.03 mg/kg. No fewer than 97 pesticide residues were found in the rose samples (on average 13.56 active substances per sample). For chrysanthemums and gerberas, pesticide residues detected were lower: an average of 6.25 active substances per chrysanthemum sample and an average of 4.35 active substances per gerbera sample with an average total load of 1.70 mg/kg. Statistical analysis confirmed that the roses were very significantly more contaminated than gerberas and chrysanthemums. The cumulated total of all the residues was as high as 97.03 mg/kg for a single bouquet of five Belgian roses. Clearly, the largest number of different a.s. and the highest total concentration of residues were detected on the rose samples.

All detected active substances are insecticides (50%) and fungicides (46%), except four growth regulators and one herbicide. The most frequently detected substances are the fungicides fluopyram (42 samples out of 90), dodemorph, propamocarb, and procymidone and their residues reached the highest concentrations on the rose samples (e.g., 41.9 mg/kg for dodemorph). Nevertheless, a certain number of the active substances detected are highly acutely toxic (acephate, methiocarb, monocrotophos, methomyl, deltamethrin, etc.) and can generate a direct effect on the nervous system (e.g., in the case of handling flowers, transfer from the hands to the mouth could cause accidental poisoning and affect the florist’s health). Even if pesticides are generally less toxic by dermal contact than by the oral route, people who handle a large number of contaminated flowers daily are exposed via dermal absorption, especially in the case of fat-soluble pesticides, and subjected to long term effects on their health. In the study of published by Morse et al. in 1979 [4], the insecticide monocrotophos was also one of the most important contaminants (detected in nine of 105 lots), with residue levels from 7.7 up to 4750 mg/kg. Other toxic insecticides (such as endosulfan and diazinon) were also frequently detected. Nevertheless, the comparison between active substances detected on flowers in both studies is poorly relevant as many new active substances are used by growers today with lower dosages.

From the survey of 25 Belgian florists it is concluded that florists may be exposed to residual deposits from contaminated flowers, especially when preparing bouquets. Contact with foliage may deposit residues onto the skin of a worker. The exposure is assumed to depend on the task duration (h/day) [33]. The length of florists’ exposure varies greatly within the year, but remains high regardless of the season (the working day varies from 2 to 6 h). The task duration of florists, which is an important factor to consider when building exposure scenarios for a specific group of workers, is lower than the default value for time of exposure (8 h) in the EFSA Guidance Document 2014 [24]. However, bad habits (eating, drinking, or smoking at work) and the absence of wearing personal protective equipment of most of the florists could increase the risk of contact with the pesticide residues.

Regarding the effect of residues on the florists’ health in Belgium, it was not possible to conclude only on the basis of personal feelings and declarations. The Belgian florists are not directly involved with pesticide handling and spraying. However, analytical results show that they can be exposed to high levels of residues during handling. According to their answers in the survey, they seem to be mainly affected by skin allergy problems. Only one had mentioned headaches and recurrent tiredness. Those observations are consistent with their usual professional practices and toxicological properties of the compounds (see Table 7 and Table 8). The survey of Lu in 2005 [11] has shown that frequent contact with residues of pesticide applied on flowers can generate detrimental health effects: workers who re-entered a recently sprayed area were 20 times more likely to get ill than those who did not. Moreover, Abell et al. [20] demonstrated in 2000 that male fecundity could be decreased after exposure to pesticides in the manual handling of ornamental flowers in greenhouses.

## 5. Conclusions

In summary, overall the samples of cut flowers (roses, gerberas, and chrysanthemums) sold in Belgium contain high pesticide residue levels. Thus, florists who handle a large number of flowers are exposed daily, with a potential effect on their health. Therefore, to reduce the exposure of florists to pesticide residues, sensitisation of professionals to better practices and hygiene rules is highly recommended. The European Regulation on Maximum Residue Limits (Regulation (EC) N° 396/2005) could be extended to the control of pesticide residues on flowers and MRLs (Maximum Residue Limits) could be set up for flowers to decrease the risk of exposure of florists and the general population. This survey will be completed later by results of field and laboratory trials to measure the dislodgeable foliar residues (DFR, µg/cm^2^), the transfer from plant to hands and, finally, to estimate the dermal exposure of florists to pesticides applied on cut flowers.

## Figures and Tables

**Table 1:** Pesticide residue levels in 90 samples of cut flowers sampled in Belgium (2016).

Total Pesticide Residues Concentration (mg/kg, All a.s. Together)	Samples with Pesticide Residues	
	Number of Samples	%
0.01–0.99	15	17
1.00–4.99	21	23
5.00–9.99	15	17
10.0–50.00	35	39
>50.00	4	4
Total	90	100

**Table 2:** Total number of active substances (a.s.) detected, average number of a.s. per sample (min-max), average total concentration of residues (mg/kg), median concentration, and maximum cumulated deposit (sample with the highest total amount of pesticide residues, in mg/kg) observed on a bouquet, for the three species.

Flower Species	Roses	Gerberas	Chrysanthemums
Total number of active substances detected	97	30	31
Average number of active substances/sample	13.6	4.3	6.2
(minimum–maximum number)	(3–28)	(1–9)	(0–15)
Total load average in pesticides/sample (mg/kg)	26.03	1.70	3.99
Median concentration/sample (mg/kg)	24.35	1.73	2.65
Maximum cumulated deposit/sample (mg/kg)	97.03	4.41	15.73

**Table 3:** Statistical analysis (Student’s *t*-test, using Minitab^®^ 16 software) of the contamination levels (number of a.s. found and the total load average in pesticides per sample) and comparison between the three species.

Flower Species	Number of Active Substances		Total Load in Pesticides (mg/kg)	
	T-Value	*p*-Value	T-Value	*p*-Value
Roses/Gerberas	4.66 ^a^	0.000	4.92 ^a^	0.000
Roses/Chrysanthemums	3.42 ^a^	0.002	4.42 ^a^	0.000
Gerberas/Chrysanthemums	−2.04 ^a^	0.050	−2.36 ^a^	0.028

^a^ Significant difference between results.

**Table 4:** Number of different active substances present in the samples of each species, according to country of origin (*n* = number of samples collected/origin). A total of 107 a.s. have been detected on samples.

Origin	Roses	Gerberas	Chrysanthemums
Belgium	38 (*n* = 8)	18 (*n* = 11)	17 (*n* = 2)
Colombia	24 (*n* = 2)	-	-
Ecuador	60 (*n* = 9)	-	-
Ethiopia	29 (*n* = 3)	-	-
Germany	22 (*n* = 1)	-	-
Israel	27 (*n* = 2)	-	-
The Netherlands	54 (*n* = 11)	24 (*n* = 9)	28 (*n* = 18)
Kenya	48 (*n* = 9)	-	-
Unknown (supermarkets)	36 (*n* = 5)	-	-

**Table 5:** Number of active substances found in the 90 samples according to their biological activity.

Biological Activity	Roses	Gerbera	Chrysanthemums
Fungicides	46	15	12
Herbicides	1	-	-
Insecticides	47	14	19
Growth regulators	3	1	-

**Table 6:** Alphabetic classification of all a.s. present in the 90 samples of roses, gerberas, and chrysanthemums, number of detections (concentrations > LOQ), frequency (samples in % containing the a.s.), and maximum concentration values.

Active Substances Detected in the Samples	Roses		Gerberas		Chrysanthemums	
	Number of Detections (out of 50) (Frequency)	Maximum Concentration (mg/kg)	Number of detections (out of 20) (Frequency)	Maximum Concentration (mg/kg)	Number of Detections (out of 20) (Frequency)	Maximum Concentration (mg/kg)
6-Benzyladenine	1 (2%)	0.02	0	<0.01	0	<0.01
Acephate	15 (30%)	21.90	0	<0.01	2 (10%)	2.10
Acetamiprid	12 (24%)	0.71	1 (5%)	0.01	0	<0.01
Acrinatrin	1 (2%)	0.05	0	<0.01	0	<0.01
Ametoctradin	6 (12%)	0.30	0	<0.01	0	<0.01
Azadirachtine	0	<0.01	3 (15%)	0.13	4 (20%)	1.30
Azoxystrobin	6 (12%)	0.06	0	<0.01	0	<0.01
Benalaxyl	1 (2%)	0.14	0	<0.01	0	<0.01
Benomyl (carbendazim)	22 (44%)	27.30	2 (10%)	0.03	0	<0.01
Bifenazate	2 (4%)	0.12	0	<0.01	17 (85%)	0.53
Bifenthrin	1 (2%)	0.69	0	<0.01	0	<0.01
Bitertanol	1 (2%)	0.03	1 (5%)	0.06	0	<0.01
Boscalid	20 (40%)	12.90	2 (10%)	0.08	1 (5%)	0.05
Bupirimate	9 (18%)	1.80	3 (15%)	0.04	0	<0.01
Buprofezin	3 (6%)	0.69	0	<0.01	0	<0.01
Carbosulfan	0	<0.01	0	<0.01	1 (5%)	0.14
Carboxin	1 (2%)	0.03	0	<0.01	0	<0.01
Chlorantraniliprole	3 (6%)	0.03	2 (10%)	0.02	0	<0.01
Chlorfenapyr	2 (4%)	0.04	0	<0.01	0	<0.01
Chloridazon	1 (2%)	0.02	0	<0.01	0	<0.01
Chlorothalonil	3 (6%)	0.12	1 (5%)	2.00	3 (15%)	3.50
Chlorpyrifos	0	<0.01	0	<0.01	2 (10%)	0.31
Clofentezine	12 (24%)	15.30	0	<0.01	0	<0.01
Cyflufenamid	1 (2%)	0.01	0	<0.01	0	<0.01
Cyfluthrin	3 (6%)	0.39	0	<0.01	0	<0.01
Cyhalothrin	6 (12%)	2.40	0	<0.01	0	<0.01
Cypermethrin	6 (12%)	0.92	0	<0.01	0	<0.01
Cyprodinil	31 (62%)	7.40	0	<0.01	0	<0.01
Deltamethrin	1 (2%)	0.22	0	<0.01	6 (30%)	1.30
Diazinon	2 (4%)	0.05	0	<0.01	0	<0.01
Dicofol	1 (2%)	1.00	0	<0.01	0	<0.01
Difenoconazole	4 (8%)	0.02	0	<0.01	0	<0.01
Dimethoate	2 (4%)	0.33	0	<0.01	0	<0.01
Dimethomorph	17 (34%)	1.90	0	<0.01	0	<0.01
Dinotefuran	2 (4%)	2.10	0	<0.01	0	<0.01
Dodemorph	37 (74%)	41.90	2 (10%)	0.02	0	<0.01
Ethirimol	13 (26%)	0.36	0	<0.01	0	<0.01
Etoxazole	3 (6%)	1.20	0	<0.01	3 (15%)	1.60
Etridiazole	0	<0.05	0	<0.05	7 (35%)	3.50
Famoxadone	11 (22%)	3.30	1 (5%)	0.04	0	<0.01
Fenamidone	5 (10%)	1.10	1 (5%)	0.02	0	<0.01
Fenamiphos	1 (2%)	3.30	0	<0.01	0	<0.01
Fenarimol	1 (2%)	0.03	0	<0.01	0	<0.01
Fenhexamid	13 (26%)	19.50	0	<0.01	2 (10%)	0.90
Fenpropathrin	0	<0.01	1 (5%)	0.02	0	<0.01
Fenpropidin	2 (4%)	0.02	0	<0.01	0	<0.01
Fensulfothion-Oxon	1 (2%)	0.02	0	<0.01	0	<0.01
Fenvalerate	1 (2%)	0.06	0	<0.01	5 (25%)	1.90
Fipronil	7 (14%)	0.68	0	<0.005	1 (5%)	0.75
Flonicamid	18 (36%)	1.40	11 (55%)	3.30	4 (20%)	0.45
Flubendiamide	3 (6%)	0.28	0	<0.01	0	<0.01
Fludioxonil	19 (38%)	2.00	1 (5%)	0.03	1 (5%)	0.02
Flufenoxuron	1 (2%)	0.02	0	<0.01	0	<0.01
Fluopicolide	15 (30%)	1.60	0	<0.01	0	<0.01
Fluopyram	23 (46%)	12.40	15 (75%)	3.00	4 (20%)	6.40
Forchlorfenuron	1 (2%)	0.19	0	<0.01	0	<0.01
Fosthiazate	1 (2%)	0.02	0	<0.01	0	<0.01
Furalaxyl	2 (4%)	9.90	0	<0.01	0	<0.01
Hexythiazox	3 (6%)	0.16	0	<0.01	0	<0.01
Imidacloprid	21 (42%)	3.00	0	<0.01	3 (15%)	0.93
Indoxacarb	3 (6%)	1.20	2 (10%)	0.16	0	<0.01
Iprodione	20 (40%)	17.40	7 (35%)	0.65	0	<0.01
Iprovalicarb	5 (10%)	5.40	0	<0.01	0	<0.01
Isocarbofos	1 (2%)	0.01	0	<0.01	0	<0.01
Kresoxim-methyl	9 (18%)	1.40	0	<0.01	0	<0.01
Lufenuron	12 (24%)	1.90	0	<0.02	5 (25%)	0.87
Mandipropamid	5 (10%)	6.70	1 (5%)	0.01	1 (5%)	0.02
Mepanipyrim	2 (4%)	5.20	0	<0.01	0	<0.01
Metalaxyl and Metalaxyl-M	5 (10%)	0.29	0	<0.01	1 (5%)	0.02
Methamidophos	14 (28%)	5.40	0	<0.01	1 (5%)	0.57
Methiocarb	1 (2%)	13.60	0	<0.01	4 (20%)	6.00
Methomyl and Thiodicarb	3 (6%)	4.50	0	<0.01	0	<0.01
Methoxyfenozide	9 (18%)	5.20	0	<0.01	1 (5%)	0.02
Metrafenone	5 (10%)	10.30	0	<0.01	0	<0.01
Myclobutanil	1 (2%)	0.13	0	<0.01	0	<0.01
Novaluron	6 (12%)	2.20	0	<0.01	0	<0.01
Oxadixyl	0	<0.01	0	<0.01	2 (10%)	0.03
Oxamyl	1 (2%)	0.01	0	<0.01	0	<0.01
Oxycarboxin	3 (6%)	0.11	0	<0.01	0	<0.01
Paclobutrazol	0	<0.01	1 (5%)	0.01	0	<0.01
Picoxystrobin	2 (4%)	1.80	0	<0.01	0	<0.01
Piperonyl-butoxide	0	<0.01	1 (5%)	0.27	4 (20%)	0.07
Pirimicarb	10 (20%)	0.26	0	<0.01	0	<0.01
Prochloraz	4 (8%)	3.10	0	<0.01	0	<0.01
Procymidone	18 (36%)	35.30	1 (5%)	0.35	0	<0.01
Propamocarb	22 (44%)	35.40	4 (20%)	0.16	0	<0.01
Pymetrozine	6 (12%)	0.56	0	<0.01	1 (5%)	0.03
Pyraclostrobin	7 (14%)	1.30	1 (5%)	0.02	0	<0.01
Pyridaben	1 (2%)	0.08	0	<0.01	0	<0.01
Pyridalyl	1 (2%)	0.01	0	<0.01	0	<0.01
Pyrimethanil	23 (46%)	13.70	0	<0.01	0	<0.01
Quinalphos	1 (2%)	0.05	0	<0.01	0	<0.01
Spinetoram	5 (10%)	0.13	0	<0.01	0	<0.01
Spinosad	9 (18%)	0.58	3 (15%)	0.40	0	<0.01
Spirotetramat	1 (2%)	0.03	3 (15%)	2.30	2 (10%)	0.10
Spiroxamine	34 (68%)	15.00	1 (5%)	0.03	1 (5%)	0.02
Tebuconazole	4 (8%)	5.20	0	<0.01	0	<0.01
Tetradifon	1 (2%)	0.08	0	<0.01	0	<0.01
Thiabendazole	1 (2%)	4.20	0	<0.01	0	<0.01
Thiacloprid	7 (14%)	5.80	0	<0.01	0	<0.01
Thiamethoxam	8 (16%)	4.20	1 (5%)	0.80	17 (85%)	2.20
Thiophanate-methyl	1 (2%)	9.90	2 (10%)	0.02	0	<0.01
Tolclofos-methyl	0	<0.01	0	<0.01	17 (85%)	5.60
Trichlofron	0	<0.02	6 (30%)	0.05	0	<0.02
Trifloxystrobin	2 (4%)	0.09	0	<0.01	1 (5%)	0.03
Triflumizole	3 (6%)	0.54	4 (20%)	0.24	0	<0.01
Triforine	1 (2%)	0.79	0	<0.01	0	<0.01

**Table 7:** Number of a.s. detected on cut flowers belonging to each category of acute toxicity hazard for the dermal route of exposure (CLP classification) [26,27,28,29,30,31].

Categories	LD_50_ (mg/kg bw)	Hazard Wording	Roses	Gerberas	Chrysanthemums
1	(0–50)	Fatal in contact with skin	2	-	-
2	(50–200)	Fatal in contact with skin	1	-	-
3	(200–1000)	Toxic in contact with skin	3	1	1
4	(1000–2000)	Harmful in contact with skin	5	2	2

**Table 8:** Number of active substances detected on the various cut flower species classified in each hazard category according to CLP regulation (with the corresponding code of hazard (only relevant categories for florist exposure are listed) [32].

Category	Code	Roses	Gerberas	Chrysanthemums
*Acute toxicity*				
Category 1	H310: Fatal in contact with skin	2	-	-
Category 2	H300: Fatal if swallowed	10	-	2
	H330: Fatal if inhaled	6	2	3
Category 3	H301: Toxic if swallowed	7	2	4
	H311: Toxic in contact with skin	2	-	2
	H331: Toxic if inhaled	10	1	3
Category 4	H302: Harmful if swallowed	21	7	9
	H312: Harmful in contact with skin	7	2	2
	H332: Harmful if inhaled	3	4	1
*Carcinogenicity*				
Category 2	H351:Suspected of causing cancer	13	5	4
*Serious eye damage/eye irritation*				
Category 1	H318: Causes serious eye damage	2	1	2
Category 2	H319: Causes serious eye irritation	3	1	1
*Germ cell mutagenicity*				
Category 1, 1A or 1B	H340: May cause genetic defects	1	1	-
Category 2	H341: Suspected of causing genetic defects	1	1	-
*Reproductive toxicity*				
Category 1, 1A or 1B	H360: May damage fertility or the unborn child	3	3	-
Category 2	H361: Suspected of damaging fertility or the unborn child	11	5	3
Additional category for effects on or via lactation	H362: May cause harm to breast-fed children	2	-	-
*Sensitisation of the respiratory tract or the skin*				
Respiratory sensitizersCategory 1, 1A or 1B	H334: May cause allergy or asthma symptoms or breathing difficulties if inhaled	1	-	1
Skin sensitizersCategory 1, 1A or 1B	H317: May cause an allergic skin reaction	21	13	11
*Skin corrosion/irritation*				
Category 1, 1A or 1B	H314: Causes severe skin burns and eye damage	1	1	-
Category 2	H315: Causes skin irritation	6	3	1
*Specific target organ toxicity (single exposure)*				
Category 3	H335: May cause respiratory irritation	4	3	2
*Specific target organ toxicity (repeated exposure)*				
Category 1	H372: Causes damage to organs through prolonged or repeated exposure	2	1	1
Category 2	H373: May cause damage to organs through prolonged or repeated exposure	7	3	3

**Table 9:** Number of active substances detected on the three species of cut flowers classified according to their AOEL values (Source: EU Pesticides Database 2015, European Commission/DGSANCO, Regulation (EC) 1107/2009) [34].

AOEL Values (mg/kg bw/day)	Roses	Gerberas	Chrysanthemums
(0.001–0.01)	19	3	6
(0.01–0.1)	43	15	13
(0.1–1)	18	9	7
>1	1	-	-
No AOEL *	16	3	5

* Active substances which have no AOEL values; not assessed at the European level.

## References

[1] Palma A.M., Ward R.W. (2010). Measuring demand factors influencing market penetration and buying frequency for flowers in the US. Int. Food Agribus. Manag. Rev..

[2] Rikken M. Le Marché Européen des Fleurs et Plantes Équitables et Durables (The European market for Equitable and Sustainable Flowers and Plants). http://www.befair.be/sites/default/files/all-files/brochure/Le%20march%C3%A9%20europ%C3%A9en%20des%20fleurs%20et%20plantes%20%C3%A9quitables%20et%20dur%E2%80%A6_0.pdf.

[3] Val’hor (2013). Croissance & Perspectives du Marché de la Fleur Coupée en Europe, No. 44 (Growth and Prospects of the Cut Flower Market in Europe, in Search of Green, No. 44).

[4] Morse D.L., Baker E.L., Landrigan P.J. (1979). Cut flowers: A potential pesticide hazard. Am. J. Public Health.

[5] Kendirli B., Çakmak B. (2007). Economics of cut flower production in greenhouses: Case study from Turkey. Agric. J..

[6] Illing H.P.A. (1997). Is working in greenhouses healthy? Evidence concerning the toxic risks that might affect greenhouse workers. Occup. Med..

[7] Das R., Steege A., Baron S., Beckman J., Harrison R. (2001). Pesticide-related illness among migrant farm workers in the United States. Int. J. Occup. Environ. Health.

[8] Penagos H., Ruepert C., Partanen T., Wesseling C. (2004). Pesticide patch test series for the assessment of allergic contact dermatitis among banana plantation workers in panama. Dermat. Contact Atop. Occup. Drug.

[9] Farahat T.M., Abdelrasoul G.M., Amr M.M., Shebl M.M., Farahat F.M., Anger W.K. (2003). Neurobehavioural effects among workers occupationally exposed to organophosphorous pesticides. Occup. Environ. Med..

[10] Alavanja M.C., Hoppin J.A., Kamel F. (2004). Health Effects of Chronic Pesticide Exposure: Cancer and Neurotoxicity* 3. Annu. Rev. Public Health.

[11] Lu J.L. (2005). Risk factors to pesticide exposure and associated health symptoms among cut-flower farmers. Int. J. Environ. Health Res..

[12] Wesseling C., De Joode B.V.W., Keifer M., London L., Mergler D., Stallones L. (2010). Symptoms of psychological distress and suicidal ideation among banana workers with a history of poisoning by organophosphate or n-methyl carbamate pesticides. Occup. Environ. Med..

[13] Baldi I., Gruber A., Rondeau V., Lebailly P., Brochard P., Fabrigoule C. (2011). Neurobehavioral effects of long-term exposure to pesticides: Results from the 4-year follow-up of the PHYTONER Study. Occup. Environ. Med..

[14] Alavanja M.C., Samanic C., Dosemeci M., Lubin J., Tarone R., Lynch C.F., Coble J. (2003). Use of agricultural pesticides and prostate cancer risk in the Agricultural Health Study cohort. Am. J. Epidemiol..

[15] Bassil K.L., Vakil C., Sanborn M., Cole D.C., Kaur J.S., Kerr K.J. (2007). Cancer health effects of pesticides Systematic review. Can. Fam. Physician.

[16] Abu M.T. (2004). Adverse impact of insecticides on the health of Palestinian farm workers in the Gaza Strip: A hematologic biomarker study. Int. J. Occup. Environ. Health.

[17] Del Prado-Lu J.L. (2007). Pesticide exposure, risk factors and health problems among cutflower farmers: A cross sectional study. J. Occup. Med. Toxicol..

[18] Lacasaña M., López-Flores I., Rodríguez-Barranco M., Aguilar-Garduño C., Blanco-Muñoz J., Pérez-Méndez O., Cebrian M.E. (2010). Association between organophosphate pesticides exposure and thyroid hormones in floriculture workers. Toxicol. Appl. Pharmacol..

[19] Gómez-Arroyo S., Díaz-Sánchez Y., Meneses-Pérez M.A., Villalobos-Pietrini R., de León-Rodríguez J. (2000). Cytogenetic biomonitoring in a Mexican floriculture worker group exposed to pesticides. Mutat. Res..

[20] Abell A., Ernst E., Bonde J.P. (2000). Semen quality and sexual hormones in greenhouse workers. Scand. J. Work Environ. Health.

[21] Hanke W., Jurewicz J. (2004). The risk of adverse reproductive and developmental disorders due to occupational pesticide exposure: An overview of current epidemiological evidence. Int. J. Occup. Med. Environ. Health.

[22] Restrepo M., Munoz N., Day N.E., Parra J.E., de Romero L., Nguyen-Dinh X. (1990). Prevalence of adverse reproductive outcomes in a population occupationally exposed to pesticides in Colombia. Scand. J. Work Environ. Health.

[23] Restrepo M., Muñoz N., Day N., Hernandez C., Blettner M., Giraldo A. (1990). Birth defects among children born to a population occupationally exposed to pesticides in Colombia. Scand. J. Work Environ. Health.

[24] European Food Safety Authority (EFSA) (2014). Guidance on the assessment of exposure of operators, workers, residents and bystanders in risk assessment for plant protection products. EFSA J..

[25] Anastassiades M., Lehotay S.J., Štajnbaher D., Schenck F.J. (2003). Fast and easy multiresidue method employing acetonitrile extraction/partitioning and “dispersive solid-phase extraction” for the determination of pesticide residues in produce. J. AOAC Int..

[26] AGP—List of Pesticides Evaluated by JMPS and JMPR. www.fao.org/agriculture/crops/thematic-sitemap/theme/pests/lpe/en/.

[27] FAO FAO Specifications for Agricultural Pesticides in Agriculture. http://www.fao.org/agriculture/crops/thematic-sitemap/theme/pests/jmps/ps-new/en/.

[28] FAO FAO Plant Production and Protection Paper Series. www.who.int/foodsafety/publications/jmpr-reports/en/.

[29] WHO/FAO Joint Meeting on Pesticide Residues (JMPR). Monographs & Evaluations. www.inchem.org/pages/jmpr.html.

[30] Inter-Organization Programme for the Sound Management of Chemicals and World Health Organization (2010). WHO Recommended Classification of Pesticides by Hazard and Guidelines to Classification 2009.

[31] World Health Organization (2004). The WHO Recommended Classification of Pesticides by Hazard and Guidelines to Classification 2004.

[32] European Commission (2009). Regulation (EC) No 1272/2008 of the European Parliament and of the Council of 16 December 2008.

[33] European Food Safety Authority (EFSA) (2010). Scientific Opinion on Preparation of a Guidance Document on Pesticide Exposure Assessment for Workers, Operators, Bystanders and Residents. EFSA J..

[34] EU—Pesticides Database. ec.europa.eu/food/plant/pesticides/eu-pesticides-database/public/?event=homepage&language=EN.

[35] Agence Fédérale pour la Sécurité de la Chaîne Alimentaire (AFSCA) (2015). Faits et Chiffres. Rapport D’activité 2015.

